# The Critical Role Played by Mitochondrial MITF Serine 73 Phosphorylation in Immunologically Activated Mast Cells

**DOI:** 10.3390/cells11030589

**Published:** 2022-02-08

**Authors:** Lakshmi Bhargavi Paruchuru, Sharmila Govindaraj, Ehud Razin

**Affiliations:** Department of Biochemistry and Molecular Biology, Institute for Medical Research Israel-Canada, Faculty of Medicine, Hebrew University of Jerusalem, Jerusalem 91120, Israel; bhargavi.lakshmi@mail.huji.ac.il (L.B.P.); sharmila.govindaraj@mail.huji.ac.il (S.G.)

**Keywords:** allergy, mast cells, mitochondria, microphthalmia-associated transcription factor, pyruvate dehydrogenase, extracellular signal-regulated kinase, degranulation, cytokines

## Abstract

In recent years, growing evidence has indicated the pivotal role of mitochondria in mast cell immunological activation. We have previously reported a decrease in degranulation and cytokine secretion following the inhibition of pyruvate dehydrogenase (PDH) either by CPI-613 (PDH inhibitor/anti-cancer drug) or through its interaction with mitochondrial microphthalmia-associated transcription factor (MITF). In the present study, we further explored the role played by mitochondrial MITF in mast cell exocytosis using rat basophil leukemia cells [RBL], as well as mouse bone marrow-derived mast cells (BMMCs). Here, we report that mast cell degranulation, cytokine secretion and oxidative phosphorylation (OXPHOS) activities were associated with phosphorylation of Serine 73 of mitochondrial MITF, controlled by extracellular signals regulated by protein kinase (ERK1/2) activity. Also, we report here that decreased OXPHOS activity following ERK1/2 inhibition (U0126 treatment) during IgE-Ag activation was mediated by the dephosphorylation of Serine 73 mitochondrial MITF, which inhibited its association with PDH. This led to a reduction in mast cell reactivity. In addition, a phosphorylation-mimicking mitochondrial MITF-S73D positively regulated the mitochondrial activity, thereby supporting mast cell degranulation. Thus, the present research findings highlight the prominence of mitochondrial MITF Serine 73 phosphorylation in immunologically activated mast cells.

## 1. Introduction

We recently reported that pyruvate dehydrogenase (PDH), a key player in oxidative phosphorylation and cellular respiration, regulates Mast Cell function controlled by its association with mitochondrial microphthalmia-associated transcription factor (MITF) [1]. PDH phosphorylation is one of the fundamental factors determining PDH activity [2] during allergic stimulus. Inhibition of PDH activity by CPI-613, a known anti-cancer agent [3], has been shown to reduce mast cell β-hexosaminidase and cytokine release [1]. Variable factors such as post-translational modifications and protein interactions with MITF influence its transcriptional activity, regulating MITF function in a coordinated manner. So far, the evidence suggests that MITF is among the most important transcription factors involved in regulation of mitochondrial function [1] and therefore complete characterization of its mode of action is of great importance. MITF is a basic-helix-loop-helix-leucine zipper (bHLHZ) transcription factor, with a major role in mast cell growth, differentiation and maturation [4]. It has previously been observed that MITF regulates the gene expression of mast cell proteases [5,6], c-KIT [7], and granzyme B [8]. Along with mitogen-activated protein kinase (MAPK) activation, mast cell IgE-Ag stimulation activates several kinase pathways [9]. This activated signal transduction pathway causes MITF phosphorylation at Serine 73, which enhances its nuclear transcriptional activity [10]. It was previously described that in melanoma cells, MAPK-mediated phosphorylation of MITF at Serine 73 assembles the two coactivators, E1A binding protein p300 and cAMP-response element binding protein (p300/CBP), which together enhance MITF transcriptional activity [11]. Previous research on melanocytes revealed that c-KIT triggers the MAPK/ERK/p90 Rsk signaling pathway [12]. This activation causes phosphorylation at two Serine sites, 73 and 409, triggering MITF transactivation accompanied by ubiquitin-mediated degradation [12].

In the present work, we show that ERK signaling-mediated Serine 73 phosphorylation of mitochondrial MITF affected its interaction with PDH and regulated mast cell function. 

## 2. Materials and Methods

### 2.1. Antibodies

Anti-MITF-C5 for immunoprecipitation experiments was provided by Prof. David Fisher (Harvard Medical School, Boston, MA, USA). Rabbit anti-MITF-phospho-Ser73 (MITF-pS73; SAB4503940) and rabbit anti-MITF (HPA003259) were purchased from Sigma-Aldrich (St. Louis, MO, USA). Rabbit anti-p44/42 MAPK-ERK-phospho-Thr202/Tyr204 of Erk1; Thr185 and Tyr187 of Erk2 (pERK; 4370), mouse anti-p44/42 MAPK-ERK 1/2 (ERK, 4696), rabbit anti-PDHA (3205), rabbit anti-glyceraldehyde-3-phosphate dehydrogenase (GAPDH; 5174), rabbit anti-VDAC (4866) antibodies were purchased from Cell Signaling Technology (Beverly, MA, USA).

### 2.2. Chemical Inhibitor Treatment

U0126 was purchased from Sigma-Aldrich and dissolved in 100% DMSO (Sigma-Aldrich, St. Louis, MO, USA), was according to the manufacturer’s instructions, for all the in vitro experiments.

### 2.3. Cell Culture

RBL-2H3 cells were maintained in RPMI 1640 medium without sodium pyruvate or L-glutamine supplemented with 10% FBS, 2 mmol/L L-glutamine, 100 U/mL penicillin, and 100 mg/mL streptomycin (Biological Industries, Beit Haemek, Israel), as previously described [13]. Unless stated otherwise, complete medium was used for the experiments. For mitochondrial ATP calculation, galactose (5 mmol/L) containing glucose-free RPMI 1640 medium without sodium pyruvate or L-glutamine and supplemented with 10% dialyzed FBS, 2 mmol/L L-glutamine, 100 U/mL penicillin, and 100 mg/mL streptomycin (Biological Industries, Beit Haemek, Israel) was used. 

### 2.4. Bone Marrow-Derived Mast Cells (BMMCs) from Mice

Male and female C3H wild-type mice aged 6 to 8 weeks were purchased from Jackson Laboratories (Cambridge, MA, USA). Mice were fed a regular diet and given drinking water ad libitum. Experiments were approved by the Hebrew University Ethical Committee for Animal Experimentation. Mice were euthanized by gradual exposure to carbon dioxide followed by cervical dislocation for the extraction of bone marrow cells from the femur and tibia bones. Afterwards, BMMCs were cultured in RPMI conditional medium, as previously described [14,15]. Cells were generally grown for a minimum of four weeks and used when greater than 90% of the population was positive for toluidine blue staining. BMMCs and RBL cells were sensitized first with 200 ng/mL anti-DNP IgE mAb (SPE-7; D8406 Sigma-Aldrich, St. Louis, MO, USA) for 2 h, washed in PBS, and then challenged with 5 ng/mL dinitrophenyl albumin (DNP; A6661 Sigma-Aldrich, St. Louis, MO, USA) for different incubation periods (5 to 60 min) depending on the experiment.

### 2.5. Cytosol and Mitochondrial Fractionation

RBL and BMMC cells were washed once in cold PBS before homogenization. Cells (20 × 10^6^) were homogenized in buffer A (250 mmol/L sucrose, 20 mmol/L HEPES, 10 mmol/L potassium chloride, 1.5 mmol/L magnesium chloride, 1 mmol/L ethylenediaminetetraacetic acid (EDTA) and 1 mmol/L ethyleneglycol-bis-(β-aminoethylether)-N,N,N9,N9-tetraacetic acid (EGTA) (pH 7.4) supplemented with 1 mmol/L phenylmethylsulfonyl fluoride (PMSF), 0.5 mmol/L sodium orthovanadate (SOV), 15 mmol/L sodium fluoride (NaF) and 1% protease inhibitor cocktail) by passing through a 10 G syringe needle followed by a brief sonication for 3 s. The procedure was performed at 4 °C. The homogenate was centrifuged for 5 min at 2000× *g*, and the supernatant was kept. This was repeated twice, and the supernatants were combined and then centrifuged for 10 min at 2000× *g* to remove the nuclei and cell debris. The supernatant was transferred to a clean tube and centrifuged for 10 min at 14,000× *g* to pellet mitochondria. They were then washed twice, resuspended in a small volume of buffer A, and stored at −80 °C until use. The mitochondrial pellet was resuspended in buffer B (lysis buffer A, 20% glycerol) with the addition of protease inhibitor cocktail (P8340, Sigma-Aldrich, St. Louis, MO, USA) to prepare the mitochondrial protein fraction. 

### 2.6. Gel Electrophoresis and Western Blotting

RBL cells were lysed by the addition of lysis buffer (50 mmol/L Tris-HCl (pH 7.4), 1% Nonidet P-40, 0.25% sodium-deoxycholate, 150 mmol/L NaCl, 1 mmol/L EDTA) supplemented with PMSF, SOV, NaF and protease inhibitor cocktail as mentioned in Section 2.5, just before use. Cells and mitochondria were vortexed and incubated on ice for 15 min. The lysates were then centrifuged at 14,000× *g* for 15 min, and pellets were discarded. The protein concentration of each sample was determined using Bradford reagent (Sigma-Aldrich). Proteins were resolved by 10% to 15% SDS-PAGE under reduced conditions and transferred to polyvinylidene difluoride membranes (Merck Millipore, Co Cork, Ireland). The membranes were incubated in 5% fat-free skim milk or BSA in TBST buffer (10 mmol/L Tris-HCl (pH 7.4), 150 mmol/L NaCl, and 0.1% Tween 20) for 1 h and then in 5% BSA and TBST containing various dilutions of primary antibodies for 18 h at 4 °C. The membranes were washed 3 times with TBST for 5 min before and after incubation with a secondary antibody. The proteins were detected with the appropriate secondary antibody (1 h at room temperature) coupled to horseradish peroxidase-conjugated goat anti-rabbit or anti-mouse antibody and visualized by means of chemiluminescence in the Bio-Rad Gel Documentation system, according to the manufacturer’s instructions. Densitometry of the respective protein bands on the western blot pictures were quantified using the ImageJ program. 

### 2.7. Mitochondrial Targeted Plasmids

An MITF expression construct used in our earlier work (1) was amplified by means of PCR and then excised and ligated into mitochondrial targeted pCMV/myc/mito vector (pShooter) (Invitrogen-Thermo Fisher Scientific, San Diego, CA, USA) [16]. Serine at MITF-73 site was mutated either with phosphorylation mimicking aspartate (S73D) or dephosphorylation mimicking alanine (S73A) using the Q5 site-directed mutagenesis kit (New England Biolabs, Ipswich, MA, USA) on the obtained mitochondrial MITF-WT (wildtype) vector. The following two sets of primers were used to generate the mutated mitochondrial MITF-S73D or S73A from the mitochondrial MITF-WT: 

Primer set 1 for mutating S = AGC to D = GAC

MITF73D_F  CGCACCCAACGACCCTATGGCTATGCTCAC

MITF73D_R  CTGCTCCCCGGCACTGGT

Primer set 2 for mutating S = AGC to A = GCC

MITF73A_F  CGCACCCAACGCCCCTATGGCTATGCTCAC

MITF73A_R  CTGCTCCCCGGCACTGGT

### 2.8. Transfection

Amaxa Nucleofector (Amaxa, Cologne, Germany) technology was used to transfect RBL cells. For plasmid transfection, a total of 2 × 10^6^ cells and 2 mg of each plasmid was used. Briefly, cells were resuspended in 100 mL of Ingenio solution (Mirus, Madison, WI, USA), DNA (plasmid) was added, and the mixture was transferred to an electroporation cuvette. Electroporation was performed with the T-20 program for RBL cells.

### 2.9. β-Hexosaminidase Release Assay (Degranulation Assay)

RBL cells (1 × 10^4^) were immunologically activated, as described above. After IgE incubation, cells were washed 3 times with 5 mL of degranulation buffer (130 mmol/L NaCl, 5 mmol/L KCl, 5.6 mmol/L glucose, 1 mmol/L MgCl_2_, 1.2 mmol/L CaCl_2_, 10 mmol/L HEPES (pH 7.4), and 0.1% BSA) and resuspended in 200 µL of the same buffer with DNP for 30 min. β-Hexosaminidase release was determined in triplicates in a 96-well plate. Aliquots (20 mL) of supernatants and cell lysates were incubated for 30 min with 100 mL of substrate solution (1.3 mg/mL p-nitrophenyl-b-D-2-acetamido-2-deoxyglucopyranozide in 0.1 mol/L citrate, pH 4.5). The reaction was stopped by the addition of 200 µL of 0.2 mol/L glycine (pH 10.7). The plate absorbance was read in an ELISA reader at a wavelength of 405 nm. Percentage release values for each experimental condition were calculated.

### 2.10. ATP Determination

RBL cells were either treated with inhibitor or transfected as mentioned above. Twenty-four hours before the ATP assay, the growth medium was changed to a glucose-free medium (RPMI 1640 without D-glucose, sodium pyruvate, or L-glutamine; Biological Industries, Beit Haemek, Israel) supplemented as above.

Mitochondrial ATP levels were measured by using the ATPlite luminescence–based assay, according to the manufacturer’s instructions (PerkinElmer, Groningen, The Netherlands).

### 2.11. Oxygen Consumption

RBL cells were either treated with inhibitor or transfected as mentioned above. Twenty-four hours before oxygen measurement, the growth medium was changed to a glucose-free medium (as above). The oxygen consumption rate was measured by Cell Mito Stress Test Kit using the Agilent Seahorse XF96 extracellular flux analyzer (Agilent Technologies, TX, USA).

### 2.12. Measurement of Cytokines, Pyruvate Levels and PDH Activity

Mast cells were sensitized with IgE anti-DNP for 2 h, followed by 30 min incubation with DNP-BSA. During the IgE sensitization, RBL/BMMCs cells were incubated either with 20 µmol/L U0126 or with DMSO as control and degranulation and cytokine (TNF-alpha, Granzyme B) secretion were assessed by measuring β-hexosaminidase release and by ELISA, respectively. Granzyme B (88-8022-22) and TNF alpha (88-7324-22) levels were estimated using the ELISA kits from Thermo Fisher Scientific, San Diego, CA, USA. Pyruvate levels (ab65342) and PDH activity (ab109902) were estimated using the kits from Abcam (Cambridge, UK) according to the manufacturer instructions.

### 2.13. Coimmunoprecipitation

RBL cells were used in coimmunoprecipitation experiments. Cells (5–10 × 10^6^) were lysed by the addition of 400 µL of cold lysis buffer (0.01 mol/L Tris-HCl [pH 7.4], 1% deoxycholate, 1% Triton X-100, 0.1% SDS, 0.15 mol/L NaCl supplemented with PMSF, SOV, NaF and protease inhibitor cocktail as mentioned in Section 2.5). Cells were then homogenized, and their supernatants were collected after 15 min centrifugation in a microcentrifuge at 14,000× *g* at 4 °C. Recovered lysates were incubated overnight at 4 °C with anti-mouse MITF-C5. The next day, 20 µL of protein A/G plus agarose beads (sc-2003, Santa Cruz Biotechnology, TX, USA) were added to the lysate prebound to the antibody and incubated with agitation for 2 h at 4 °C. The recovered immune complexes were washed 3 times with cold PBS buffer 400 µL and 25 µL of sample buffer (3 mL of glycerol, 3 mL of 20% SDS, 1.6 mL of β-mercaptoethanol, 2.4 mL of 1 mol/L Tris-HCL (pH 6.8), and 6 mg bromophenol blue for color) was added to the prewashed beads. Beads with the sample buffer were boiled at 100 °C for 10 min and loaded on a 10% SDS gel.

### 2.14. Statistical Analysis

For the quantification of Western blots, a two-tailed Student t-test statistical analysis was made and one way-ANOVA was performed for comparing the data in other assays. Data represented as means ± SEMs.

## 3. Results

### 3.1. IgE-Ag Mast Cell Activation Induces Phosphorylation of MITF in Mitochondria

**Figure 1:**
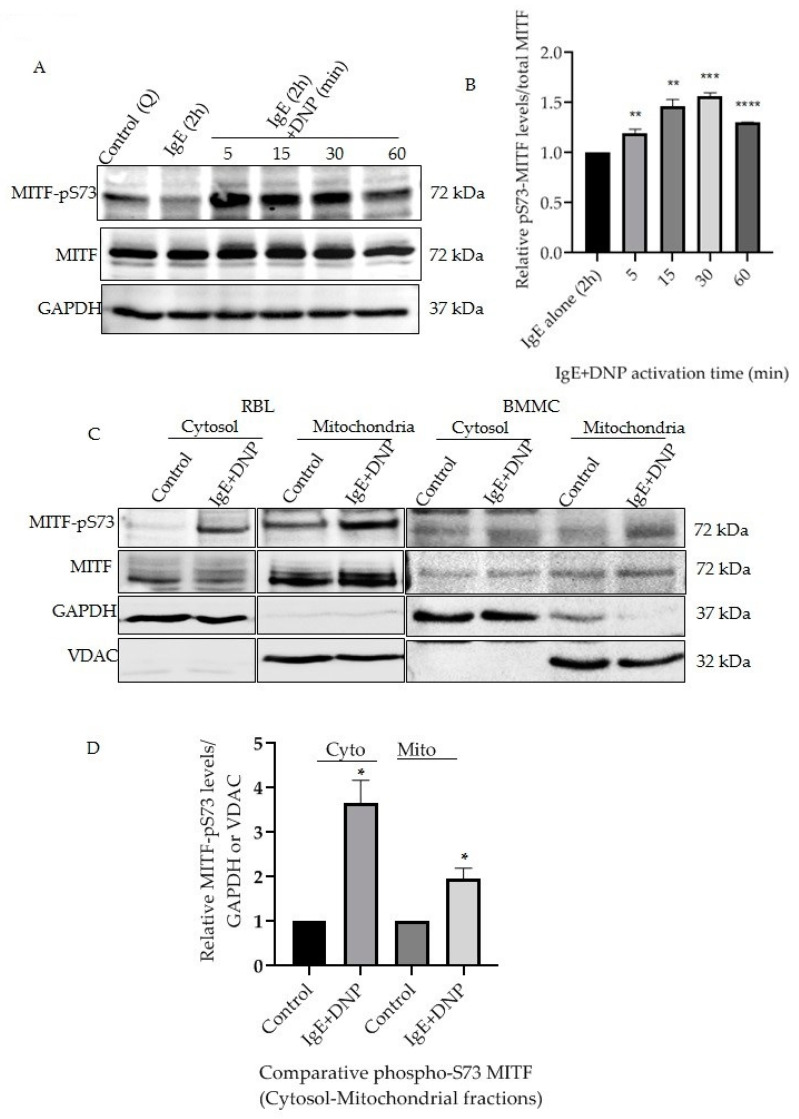
IgE-Ag stimulation affects mitochondrial MITF Serine 73 phosphorylation. (**A**), RBL cells were sensitized with 100 ng/mL IgE for 2 h followed by a 0–60 min DNP challenge. Non-sensitized cells are presented as Control (Q: Quiescent). The protein levels of MITF in the whole cell lysates were determined by Western blot analysis using anti-pS73-MITF, anti-MITF and anti-GAPDH antibodies. (**B**), Densitometry of MITF-pS73/MITF levels in RBL cells that were activated for 0–60 min. (**C**), RBL and BMMCs were treated with 100 ng/mL IgE for 2 h followed by a 15 min DNP challenge and were fractionated into cytosol and mitochondria. The protein levels of MITF in both fractions were determined by Western blot analysis using anti-pS73-MITF, anti-MITF, anti-GAPDH (cytosolic marker) and anti-VDAC (mitochondrial marker) antibodies. (**D**), Densitometry of MITF-pS73/GAPDH and MITF-pS73/VDAC levels in RBL cells that were activated for 15 min followed by cytosolic and mitochondrial fractionation. A two-tailed Student *t*-test was performed for all the western blots. Results represent means ± SEMs (n = 4, **** *p* ≤ 0.0001; *** *p* ≤ 0.001; ** *p* ≤ 0.01; * *p* ≤ 0.05; ns *p* > 0.05).

Mast cells triggered by monomeric IgE through the MAPK and AKT signaling mechanisms induce cytokine secretion and increase cell survival [17,18]. This IgE stimulation alone affects MITF Serine 73 phosphorylation and increases MITF’s nuclear transcriptional activity [10]. The present section of the study was initiated to understand whether prolonged exposure of antigen (Ag)/allergen DNP on IgE-stimulated cells has any effect on MITF phosphorylation and to identify the changes at the mitochondrial level. RBL cells were sensitized with IgE for 2 h followed by DNP challenge for 5–60 min. The cells were lysed as mentioned in Section 2.6 and Western blot analysis was done using the antibodies against MITF-pS73 and MITF. The results presented in Figure 1A clearly show that the response to the immunological trigger is time dependent, as phosphorylation of MITF at Serine 73 is induced in a time-dependent manner in samples of whole cell lysate. Densitometry analysis of MITF-pS73 relative to the respective MITF bands at different time intervals is shown in Figure 1B. In order to check the status of MITF phosphorylation localized in both cytosol and mitochondria, a fractionation methodology was followed as mentioned in Section 2.5 and Section 2.6 to visualize protein levels using western blots. A rise in phosphorylation of MITF on Serine 73 was observed in both mitochondrial and cytosolic fractions after immunological activation for 15 min, in both RBL and BMMCs (Figure 1C,D). Purity of the subcellular fractionation was confirmed using GAPDH as cytosolic marker and VDAC as mitochondrial marker; these were also the corresponding MITF loading controls (Figure 1C).

### 3.2. Mitochondrial Serine 73-MITF Is ERK1/2-Dependently Phosphorylated during Mast Cell IgE-Ag Stimulation

**Figure 2:**
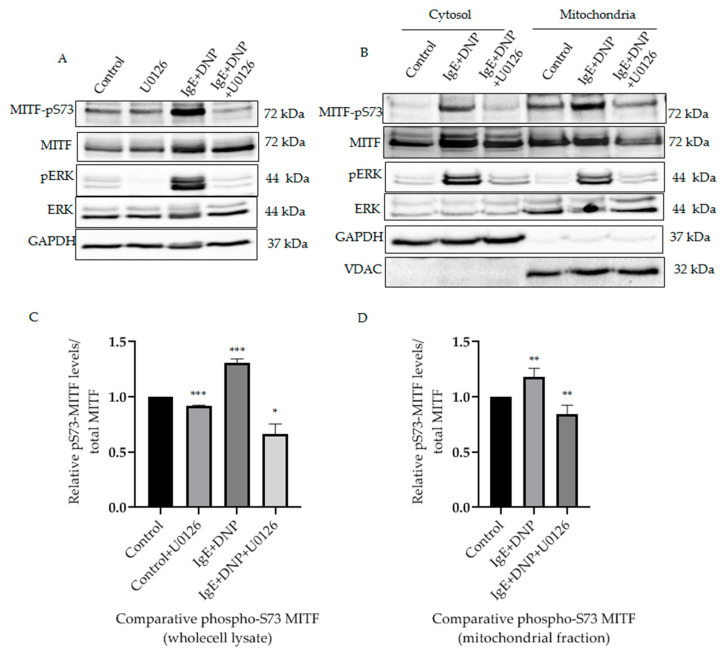
ERK1/2-dependent mitochondrial MITF Serine 73 phosphorylation in immunologically activated mast cells. (**A**), RBL cells were sensitized with 100 ng/mL IgE for 2 h, followed by a 15 min DNP challenge. Thirty minutes before the addition of DNP, 20 µmol/L U0126 was added to the medium. The protein levels of MITF and ERK in the whole cell lysates were determined by Western blot analysis using anti-pS73-MITF, anti-MITF, anti-pERK, anti-ERK and anti-GAPDH antibodies. (**B**), RBL cells were treated as in (**A**). The cells were fractionated into cytosol and mitochondria. The protein levels of MITF in both fractions were determined by Western blot analysis using anti-pS73-MITF, anti-MITF, anti-pERK, anti-ERK, anti-GAPDH (cytosolic marker) and anti-VDAC (mitochondrial marker) antibodies. (**C**,**D**), Densitometry of MITF-pS73/total MITF levels in RBL cells wholecell lysate (**C**) and mitochondrial fractions (**D**) that were treated with U0126 and DMSO as control as in (**A**). A two-tailed Student *t*-test was performed for all the western blots. Results represent means ± SEMs (n = 4, *** *p* ≤ 0.001; ** *p* ≤ 0.01; * *p* ≤ 0.05; ns *p* > 0.05).

It was known that in activated melanoma cells extracellular signaling kinases 1 and 2 (ERK1/2) regulate phosphorylation of MITF on the Serine 73 residue [11]. Yet, such phosphorylation of MITF inside the mitochondria has not been previously identified. It was important to determine that phosphorylation on S73 is carried out by a specific kinase and is not due to translocation upon IgE-Ag stimulation, since the pathological stress or external stimuli makes the protein localization and effects the mitochondrial dynamics [19,20]. Therefore, we investigated the correlation between mitochondrial ERK1/2 and MITF-Serine 73′s phosphorylation in activated mast cells. It can be seen from our results that ERK1/2 signaling phosphorylates MITF on Serine 73 during mast cell activation (Figure 2A). Following IgE-DNP RBL activation in mast cells, the level of Serine 73 phosphorylated MITF was increased in whole lysates (Figure 2A) and in the mitochondria (Figure 2B). The amount of phosphorylated ERK1/2 in the mitochondria was also increased in these activated cells (Figure 2B). An increase in ERK1/2 levels in mitochondria after immunological stimulation was reported by us in our earlier study [16]. When RBL cells were treated with U0126, an ERK1/2 pathway inhibitor [21], the pS73-MITF level decreased in whole cell lysate compared to control (DMSO alone) (Figure 2A). A similar decrease in pS73-MITF was observed in mitochondria, as can be seen in the western blot shown in Figure 2B, and in the corresponding densitometry analysis using VDAC as loading control (Figure 2C). Therefore, phosphorylation of mitochondrial MITF at Serine 73 in immunologically activated mast cells is dependent on ERK1/2 signaling.

### 3.3. Dissociation of Mitochondrial Serine 73-Phosphorylated MITF from PDH after Immunological Activation

We previously reported the association of mitochondrial MITF with PDH, whereby the association-dissociation status was dependent on immunological activation of the mast cell. PDH is known to be dephosphorylated after allergic stimulation, causing its dissociation from MITF, freeing it to play an active role in mitochondria [1]. To determine whether phosphorylated MITF interacts with PDH after immunological activation and in the presence of ERK1/2 inhibitor, immunoprecipitation experiments were performed. MITF was immunoprecipitated with MITF-specific antibody, and its interaction with PDH was determined by means of Western blot analysis with anti-PDHA.

**Figure 3:**
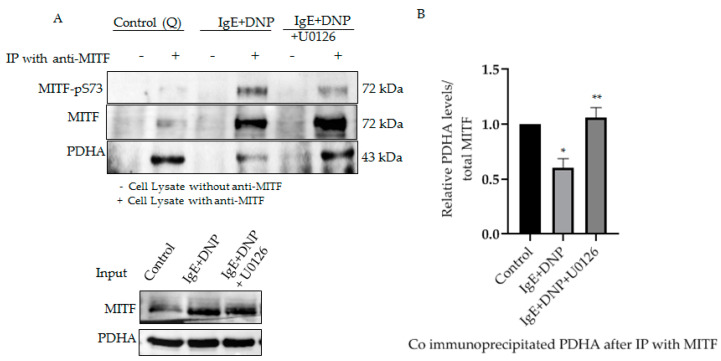
Serine 73 phosphorylation of MITF affects MITF-PDH interaction during mast cell stimulation. (**A**), IP of MITF in IgE-DNP activated RBL cell lysates in the presence and absence of ERK1/2 inhibitor. Cell lysates were pre-treated with U0126 as described in Figure 2. Non-sensitized cells are presented as Control (Q: Quiescent). For IP anti-MITF antibody was used and for Western blot analysis, anti-pS73-MITF, anti-MITF, anti-PDHA antibodies were used. (**B**), Densitometry of PDHA/MITF levels in RBL cells after IP experiment. A two-tailed Student *t*-test was performed for all the western blots. Results represent means ± SEMs (n = 4, ** *p* ≤ 0.01; * *p* ≤ 0.05; ns *p* > 0.05).

As can be seen in Figure 3, Serine 73 phosphorylation of MITF due to IgE-Ag stimulation causes PDH to dissociate from the MITF-PDH complex. However, IgE-Ag stimulation, in the presence of ERK1/2 inhibitor (U0126), results in MITF remaining associated with PDH in the MITF-PDH complex. This experiment clearly shows the significance of this phosphorylation site in the interaction between these two proteins.

### 3.4. Inhibiting Serine 73 Phosphorylation of Mitochondrial MITF during IgE-Ag Stimulation Reduces the Mast Cell Reactivity

**Figure 4:**
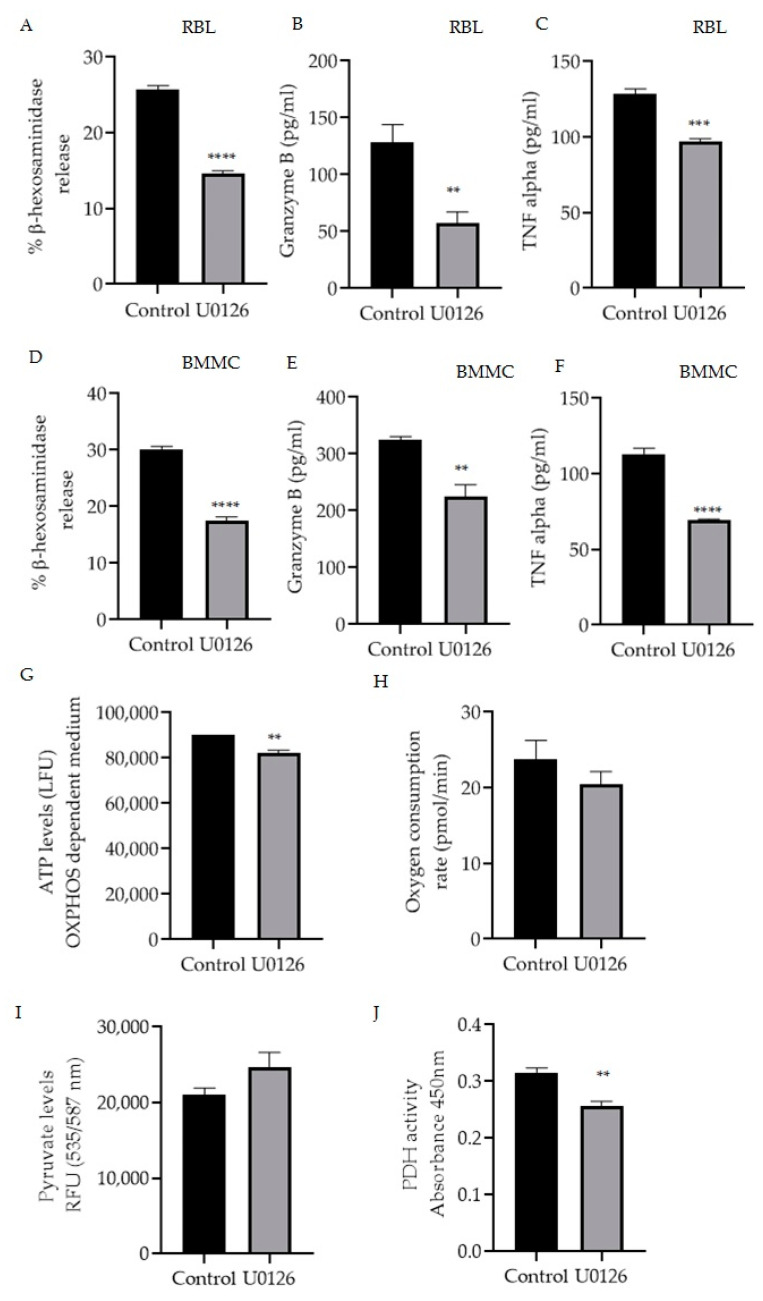
Inhibition of mitochondrial MITF Serine 73 phosphorylation using U0126 affects mast cell degranulation and mitochondrial functions. Mast cells were stimulated with IgE-DNP and incubated either with 20 µmol/L U0126 or DMSO as control and degranulation activity was assessed by measuring β-hexosaminidase release and cytokine (TNF-α, granzyme B) secretion using the ELISA assays in RBL cells (**A**–**C**) and BMMCs (**D**–**F**). Mitochondrial activity assay results are shown in (**G**) mitochondrial ATP, (**H**) Oxygen consumption rate with the increased pyruvate levels (**I**) and the decreased pyruvate dehydrogenase activity (**J**) in RBL cells. A two-tailed Student’s *t*-test was performed for all the assays. Results represent means ± SEMs (n = 4, **** *p* ≤ 0.0001; *** *p* ≤ 0.001; ** *p* ≤ 0.01; ns *p* > 0.05).

Mitochondrial MITF’s phosphorylation of Serine 73 in activated RBL mast cells was decreased by U0126 inhibitor treatment (Figure 2B). Therefore, the effect of this inhibitor on mast cell function was assessed by measuring β-hexosaminidase release, TNF-α and granzyme B levels in RBL cells (Figure 4A–C) and BMMC (Figure 4D–F). Figure 4G–J shows the effect of inhibitor treatment on ATP levels, oxygen consumption rate (OCR) in OXPHOS-dependent medium along with pyruvate and PDH activity levels.

As shown in Figure 4A,D, p-S73 MITF inhibition resulted in a significant 50% reduction in degranulation levels (n = 4, *p* < 0.0001). U0126 treatment significantly decreased the release of cytokines TNF-α in RBL cells (n = 4, *p* = 0.002) and in BMMC (n = 4, *p* < 0.0001) and granzyme B in RBL cells (n = 4, *p* = 0.0097) and in BMMC (n = 4, *p* = 0.0037). 

In order to determine the effect of U0126 on mitochondrial function, RBL cells were cultured for 24 h in a glucose-free incomplete medium that creates OXPHOS-dependent conditions supplemented with dialyzed serum as an energy source or in the complete medium as a control. Afterwards, cells were incubated with 20 µmol/L U0126 for 2 h and ATP levels were measured with the ATPlite Luminescence Assay System (Perkin Elmer). OCR was also assessed using the XF96 extracellular flux analyser (Seahorse Biosciences) As shown in Figure 4G,H U0126 inhibitor significantly reduced mitochondrial ATP (n = 4, *p* = 0.0036) with decreased oxygen levels (n = 4, *p* = 0.3166), correlating to the reduction of cytokine secretion levels. To further evaluate the effect of these inhibitors on pyruvate dehydrogenase complex activity, the levels of pyruvate and the substrate of PDH, which would be expected to accumulate if PDH is inhibited, were also checked. RBL cells were treated as described in Figure 2 using U0126, and then the total pyruvate levels were measured with a pyruvate ELISA kit. As shown in Figure 4I, inhibition of p-S73 MITF increased the pyruvate levels and a significant decrease in PDH activity (Figure 4J) was noted (n = 4, *p* < 0.001).

### 3.5. Checking the Overexpression of Phosphorylated and Dephosphorylated Mimicking Mitochondrial MITF Serine 73 on Mitochondrial Function and Mast Cell Activity

**Figure 5:**
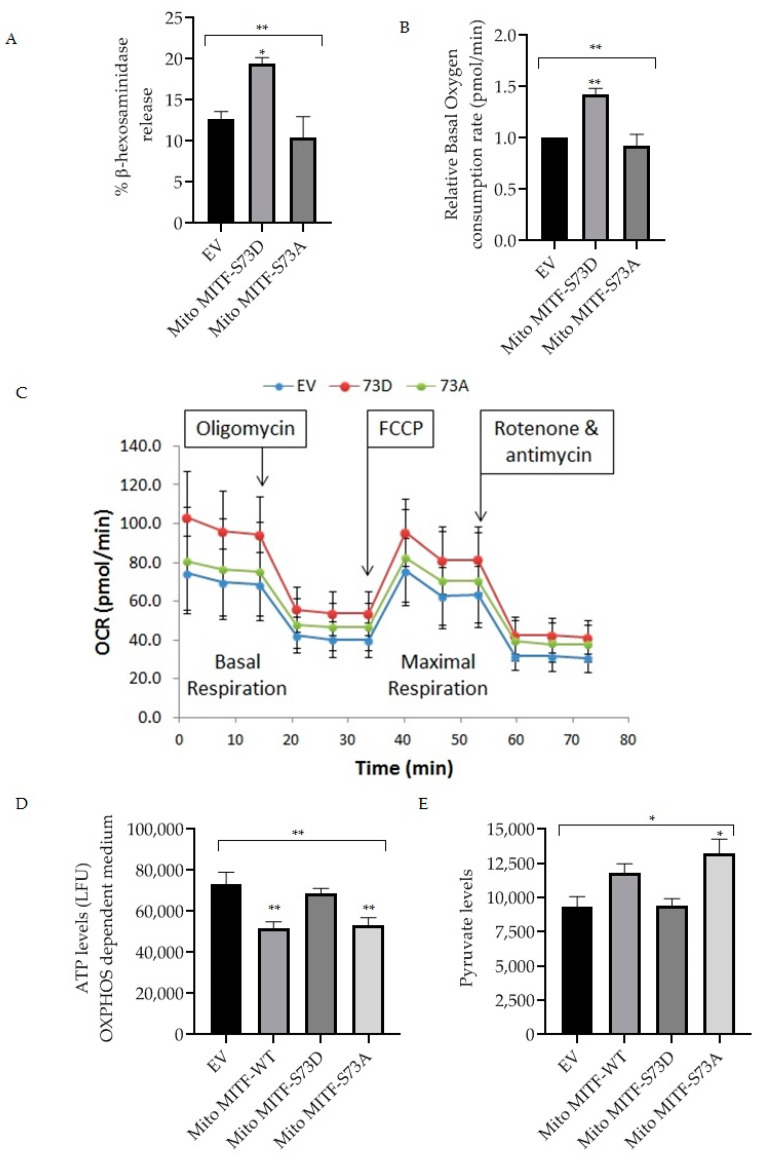
Effect of overexpression of mitochondrial MITF Serine 73 mutants (**D** or **A**) on mast cell degranulation and mitochondrial functions. Transfections were made in RBL cells either with EV or mitochondrial MITF-S73D or mitochondrial MITF-S73A. After 48 h, cells were stimulated with IgE-DNP and degranulation activity was assessed by measuring β-hexosaminidase release (**A**). Relative oxygen consumption rate at basal respiration was compared in transfected RBL cells (**B**). One (out of 3 experimental repeats from Figure 5B) of the Seahorse XF cell Mito Stress profile is shown to assess the effect of mitochondrial MITF-S73D or S73A on mitochondrial respiration, where each dot represents means ± SDs (n = 12) (**C**). Mitochondrial ATP (**D**) and pyruvate levels (**E**) 48 h after transfection were measured and compared with mitochondrial MITF-WT. Results represent means ± SEMs (n = 3 or 4, ** *p* ≤ 0.01; * *p* ≤ 0.05; ns *p* > 0.05).

Dephosphorylation of mitochondrial MITF at Serine 73 followed by U0126 inhibitor treatment decreased mast cell reactivity by lowering the PDH activity (Figure 3 and Figure 4). In order to provide support for the importance of mitochondrial MITF Serine 73, two mutant plasmids (phosphorylated and dephosphorylated mimicking) were transfected and then mast cell characteristics were analyzed. Figure 5A shows the effect of mitochondrial MITF-S73D or A on β-hexosaminidase release in RBL cells; Figure 5B shows the oxygen consumption rate and Figure 5C shows the pattern of mitochondrial respiration obtained from the Seahorse XF Mito Stress test.

As can be seen in Figure 5A, overexpression of mitochondrial MITF-S73D resulted in a significant increase in degranulation levels (n = 4, *p* = 0.0277). Mitochondrial MITF-S73A decreased the release of β-hexosaminidase by approximately 18% in RBL cells when compared with EV, where the data is statistically significant (n = 4, *p* = 0.0085).

In order to determine the effect of mutant plasmids on mitochondrial function, transfected RBL cells were cultured for 24 h in a glucose-free medium supplemented with dialyzed serum as an energy source. 48 h after transfection, oxygen consumption rate was assessed using the XF96 extracellular flux analyser. As shown in Figure 5B, mitochondrial MITF-S73D significantly increased the basal mitochondrial respiration (n = 3, *p* = 0.0099). In contrast, mitochondrial MITF-S73A decreased the oxygen levels by 8.5% when compared with EV and by 36% when compared to mitochondrial MITF-S73D, where the data is statistically significant (n = 3, *p* = 0.0052). ATP levels were measured with the ATPlite Luminescence Assay System in glucose-free medium 48 h after transfection (Figure 5D). To further evaluate the effect of these mutants on PDH activity, pyruvate levels were also checked using a pyruvate ELISA kit. As shown in Figure 5E, mitochondrial MITF-S73A had significantly higher pyruvate levels (n = 3, *p* < 0.0192) which is in correlation with the significant decrease in ATP levels (n = 4, *p* < 0.0088). Both ATP and pyruvate levels data were statistically significant in comparison with the mean of EV (n = 4, *p* < 0.0038; n = 3, *p* < 0.0191, respectively).

## 4. Discussion

Mast cells are well known for their contribution as the first line of defense in innate and adaptive immunity. The inflammatory response of mast cells can be triggered by numerous external stimuli from the environment, ranging from bacteria, insects, food, pollen, pollution and even emotional stress. Out of various mechanisms that could activate mast cells to release cytokines and other mediators into the external microenvironment, the most studied mechanism is the IgE-dependent signalling pathway. Understanding the mitochondrial homeostasis which supports mast cell activation by regulating the energy metabolism and dynamics and the functions of the newly identified regulators such as PDH, MITF and STAT3 is of great importance [20]. Our earlier studies established the fact that mast cell activation is based on mitochondrial OXPHOS activity and is regulated by transcription factors such as MITF and STAT3 [1,22]. Even though MITF’s functions as a transcription factor in nuclei are well studied, its active role in mitochondria is unexplored. The present study evaluates one of the most important post-translational modifications, i.e., phosphorylation of MITF in mitochondria via ERK1/2.

Mast cell activation by IgE-DNP leads to an increase in nuclear MITF phosphorylation on Serine 73 [10], and this phosphorylation is time dependent. MITF phosphorylation is short term and happens immediately after allergic trigger, since longer exposure time may lead to ubiquitin-mediated degradation, as was observed in previous studies in melanoma [12,23]. Subcellular fractionation of mast cells into the cytosol and mitochondria reveals that MITF is Serine 73 phosphorylated after IgE-DNP stimulus. Localization of ERK1/2 into the mitochondria indicates its active role in phosphorylating mitochondrial substrates such as the BCL2 family proteins to influence metabolism, cell survival and mitophagy [24], by which ERK 1/2 activated by phosphorylation after allergic activation maintains the energy homeostasis. To understand whether phosphorylation of MITF at Serine 73 is due to mitochondrial ERK1/2, a well-known ERK1/2 inhibitor U0126 was added during IgE-DNP stimulation of RBL cells and BMMCs. A significant decrease in pS73-MITF levels with unchanged MITF levels indicates active ERK is responsible for this phosphorylation.

The mast cell inhibitory function of U0126 in connection with OXPHOS activities by decreasing the STAT3 phosphorylation on Serine 727 denotes its potential in developing anti-allergic drugs. Our results show that U0126 causes a significant decrease in mitochondrial ATP and oxygen consumption rate. The rise in pyruvate levels with the decreased PDH activity after U0126 treatment shows the regulatory mechanism of MITF Serine 73 phosphorylation on the PDH complex. U0126 significantly reduced TNFα and granzyme B release after IgE-DNP activation when compared to control. 

Additionally, the direct influence of mitochondrial targeted MITF as a protein interactor in context to its phosphorylation state at Serine 73 was presented in this work. Even though nuclear transcription factors are best known for their major role in gene expression, recent studies on different other transcription factors like p53 [25], CREB [26] and STAT3 [27,28] translocation to mitochondria denotes their other functionality as a direct protein interactor to influence mitochondrial bioenergetics. During immunological activation of mast cells, MITF-Serine 73 phosphorylation was noted and overexpression of the phosphorylated mimicking mitochondrial MITF-S73D caused elevated degranulation compared to control cells. At the same time, mitochondrial functionality assays of oxygen, ATP, and pyruvate levels were influenced in opposing directions when mast cells were transfected with either mitochondrial MITF-S73D or S73A. This phenomenon denotes the importance of flexible post translational modifications up on external stimuli, affecting the protein-protein interactions to transform the mitochondrial functions of the activated cell [29]. 

Maintaining the mitochondrial functions through the active PDH complex converting pyruvate to acetyl COA is necessary for supporting the energy needs [30,31] of the mast cell allergic response [1]. In light of its well-established role during IgE-DNP stimulus, identifying the molecules which could alter PDH functionality is beneficial. One such regulation is MITF interaction with PDH in a quiescent state, as well as their dissociation after activation when MITF was Serine-73 phosphorylated. IP experiments in different conditions reveal the fact that active ERK1/2, when inhibited by U0126, dephosphorylates MITF at Serine 73. This inhibition makes the MITF bind to PDH, consequently affecting the PDH function. An active pyruvate dehydrogenase complex can control the metabolic changes acting as the gatekeeper by linking the glycolysis to the Krebs cycle, implicating the necessity of designing targeted therapeutic agents [32]. The summary of the present study was represented in graphical abstract. 

In conclusion, the findings of the present research indicate that MITF is Serine 73 phosphorylated by ERK1/2 in mitochondria due to activation. After IgE-DNP stimulus phosphorylated-73 Serine MITF dissociates from PDH, thereby increasing the PDH functionality for increasing the mitochondrial OXPHOS activities, supporting the degranulation and cytokine secretion of mast cells.

## Data Availability

Not applicable.
